# Impact of Vaccination on 14 High-Risk HPV Type Infections: A Mathematical Modelling Approach

**DOI:** 10.1371/journal.pone.0072088

**Published:** 2013-08-29

**Authors:** Simopekka Vänskä, Kari Auranen, Tuija Leino, Heini Salo, Pekka Nieminen, Terhi Kilpi, Petri Tiihonen, Dan Apter, Matti Lehtinen

**Affiliations:** 1 Department of Vaccination and Immune Protection, National Institute for Health and Welfare (THL), Helsinki, Finland; 2 School of Public Health, University of Tampere, Tampere, Finland; 3 Department of Obstetrics and Gynaecology, Helsinki University Central Hospital, Helsinki, Finland; 4 Sexual Health Clinic, Family Federation of Finland, Helsinki, Finland; The University of Hong Kong, Hong Kong

## Abstract

The development of high-risk human papillomavirus (hrHPV) infection to cervical cancer is a complicated process. We considered solely hrHPV infections, thus avoiding the confounding effects of disease progression, screening, and treatments. To analyse hrHPV epidemiology and to estimate the overall impact of vaccination against infections with hrHPVs, we developed a dynamic compartmental transmission model for single and multiple infections with 14 hrHPV types. The infection-related parameters were estimated using population-based sexual behaviour and hrHPV prevalence data from Finland. The analysis disclosed the important role of persistent infections in hrHPV epidemiology, provided further evidence for a significant natural immunity, and demonstrated the dependence of transmission probability estimates on the model structure. The model predicted that vaccinating girls at 80% coverage will result in a 55% reduction in the overall hrHPV prevalence and a higher 65% reduction in the prevalence of persistent hrHPV infections in females. In males, the reduction will be 42% in the hrHPV prevalence solely by the herd effect from the 80% coverage in girls. If such high coverage among girls is not reached, it is still possible to reduce the female hrHPV prevalence indirectly by the herd effect if also boys are included in the vaccination program. On the other hand, any herd effects in older unvaccinated cohorts were minor. Limiting the epidemiological model to infection yielded improved understanding of the hrHPV epidemiology and of mechanisms with which vaccination impacts on hrHPV infections.

## Introduction

The development of high-risk human papillomavirus (hrHPV) infection to cervical cancer is a complicated process including transmission and clearance of infections with different HPV types as well as progression and regression of associated lesions. Furthermore, cytological screening and treatment of lesions interfere with the natural course of disease progression and provide only incomplete information about the underlying processes. Vaccination is considered to protect against cervical cancer by preventing infections with a subset of hrHPV [1], [2]. Two currently available vaccines have shown significant protection against two target types (HPV16, HPV18), but also some protection against a number of other, up to 4 hrHPV types (HPV31, HPV33, HPV45, HPV51), and 43% to 93% efficacies against all cervical intraepithelial lesions of grade 3 (CIN3), the immediate precursor of cervical cancer [2], [3], [4], [5], [6]. The highest vaccine efficacies have been reported for the bivalent vaccine in baseline HPV-naïve group [6]. Preliminary information about the effectiveness of HPV vaccination programs can be gained from hrHPV prevalence much earlier than the cancer incidence would change. In addition, possible vaccine failures and type replacement would appear first on the infection level [7].

To understand HPV transmission and the impact of vaccination on the hrHPV infection epidemiology, it is beneficial to disentangle the infection and disease processes thus avoiding the confounding effects of screening and treatment policies. For example, the findings of precancerous lesions depend strongly on screening activity, and so, if infection-related parameters are estimated together with the disease process, the estimates are likely affected by possible errors and uncertainties in the screening model. A separate infection model facilitates the analysis of the characteristics of hrHPV infections, including natural immunity against infection and differences across hrHPV types in clearance and vaccine efficacy. An early model of HPV epidemiology separated HPV transmission and the disease process [8]. Since then epidemiological models of HPV have typically included both transmission and disease progression (e.g., [9], [10], [11], [12], [13], [14], [15])) although some have focused on the parameters of hrHPV infection [11]. In this work, we address only HPV infections.

We present a model for 14 hrHPV types, calibrated to population-based data on hrHPV prevalence in Finnish women [16] and cohort data of type-specific hrHPV infection in the control arm of the population based PATRICIA phase III vaccine study [2] in Finland. The HPV vaccination program is planned to start in Finland in autumn 2013. To the best of our knowledge, this is the first compartmental HPV transmission model that includes single and multiple infections with a large number of hrHPV types, even though corresponding micro-simulation models exist [17], [18], [19], [20]. In general, compartmental models are more suitable for analysing the role of parameter uncertainty than individual-based models as there is no stochastic variation across model outputs. An earlier model predicting the impact of HPV vaccination on cervical cancer [9] was fitted to Finnish seroprevalence data of HPV16, which approximates the cumulative incidence with moderate sensitivity. Our model was used to investigate the relative roles of the key characteristics of hrHPV epidemiology and to predict the impact of vaccination on the hrHPV infection epidemiology under different vaccination scenarios.

## Materials and Methods

**Figure 1:**
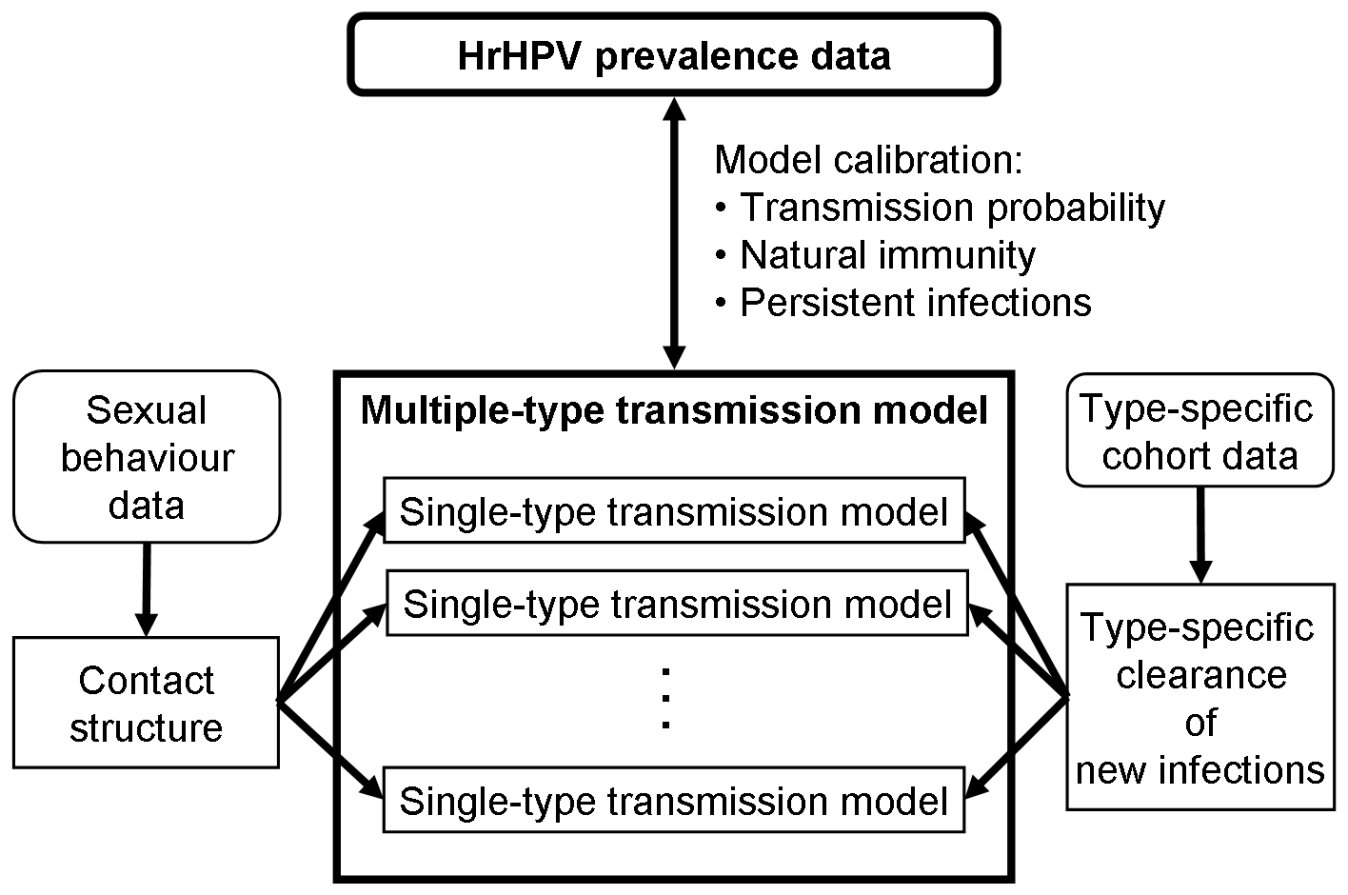
Data and Modelling Overview. The parameters of the sexual contact structure were estimated from the School Health Promotion study [21], FINSEX 2007 study [22], and marriage statistics [23]. The type-specific clearance of new infections was estimated from the control arm of PATRICIA phase III HPV vaccine study in Finland [2]. The contact structure and the type-specific clearance rates were used as input for the single-type transmission models. The multiple-type transmission model ties together the single-type models and produces the hrHPV prevalence, which was fitted to the age-specific hrHPV prevalence data [16] by calibrating three model parameters (transmission probability, natural immunity, and the clearance rate of persistent infections).

For each single hrHPV type, we considered HPV transmission in an “*SIRS*+*V*” model, dividing the population into four type-specific epidemiologic states: *S* for susceptible, *I* for infectious, *R* for recovered and *V* for vaccine-protected individuals. The population was further stratified into behavioural subpopulations. In the following, we outline the parameter sources and new modelling features, including the contact structure based on the lifetime partner number, and the construction of a multiple-type transmission model from single-type models (Figure 1). File S1 includes the detailed model specification as well as all data that were used as input in the analyses of this paper. The transmission model was programmed with MATLAB and the simulations were run on a standard laptop.

### Contact Structure

Sexual activity was assumed to depend on age, gender, and lifetime partner number. Let 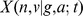 be the proportion of individuals with *n* 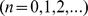 lifetime partners and vaccination status *v* (*v* = vaccinated/unvaccinated) among those of gender *g* (*g* = *f/m* = female/male) and age *a* at time *t.* Assume that the lifetime partner number in the 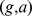 subpopulation does not depend on the vaccination status or the calendar time. Then
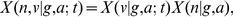
(1)where 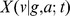 is the proportion of vaccinated in the 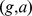 subpopulation at time *t*. The partner number distribution 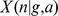 was obtained from a continuous-time Markov process with the new partner acquisition rates as the transition rates. The new partner acquisition rate 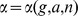 is the rate (hazard) at which an individual in the 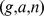 subpopulation acquires new partners. The entry age in the model was 10 years, at which age all individuals have 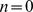 lifetime partners. Based on the partner number and vaccination status, a model for the distribution of contacts between the different subpopulations was constructed according to the age distribution of heterosexual pair formation and the proportionate mixing principle.

### Sexual Behaviour Parameters

**Figure 2:**
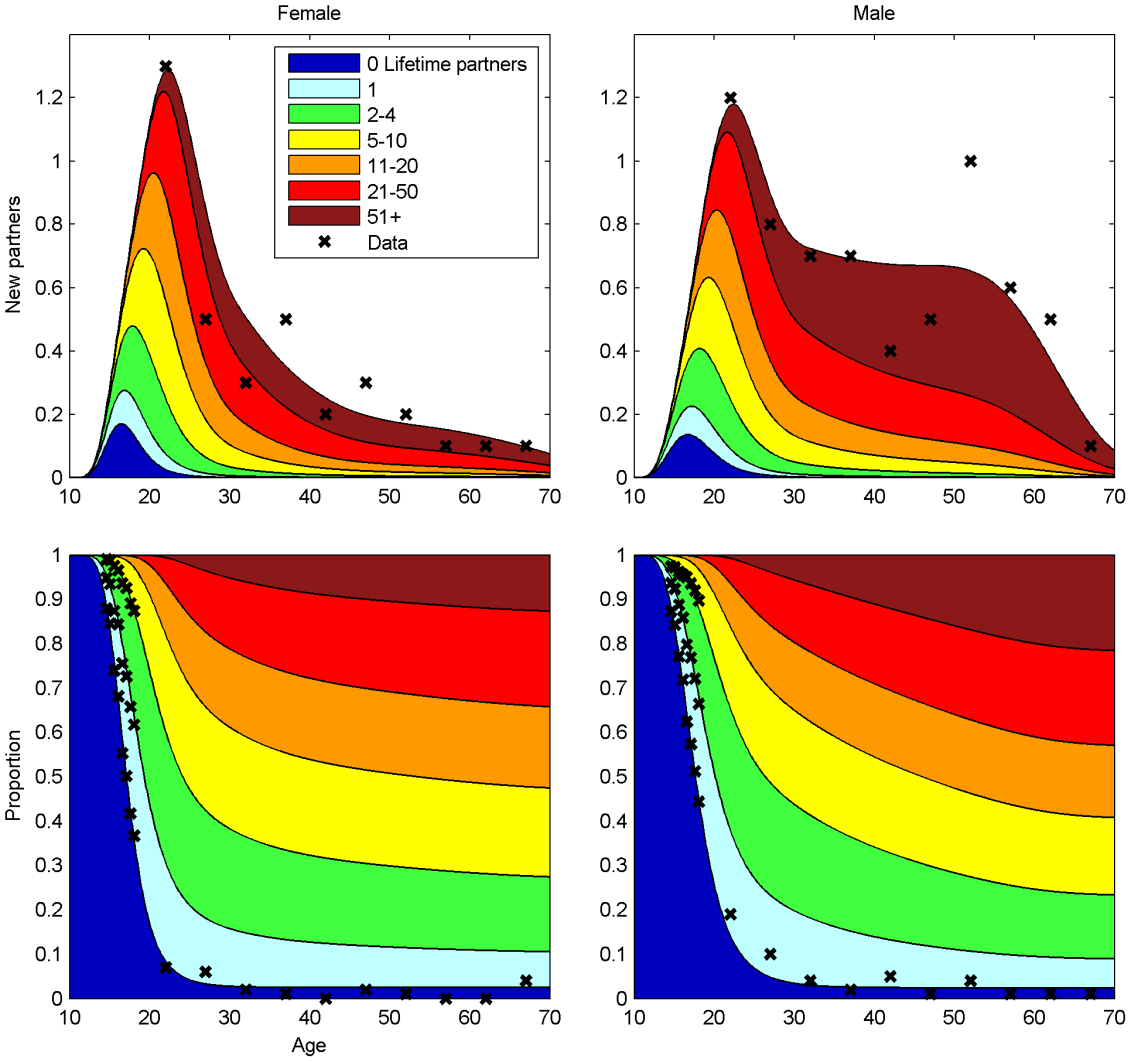
The Pattern of Sexual Contacts in Finland. Upper panel: the age-specific annual mean numbers of new sexual partners by lifetime partner number with the observed numbers (asterisks). Lower panel: the age-specific stratification of the population by lifetime partner number and the corresponding data (asterisks).

The new partner acquisition rate 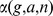 and the corresponding partner number distribution (Figure 2, Figures S1–S3 in File S1) were estimated from the School Health Promotion (SHP) Study 2008–2009 [21], the FINSEX 2007 study [22], and national data on age at marriage [23]. The biannual SHP study covers over half of the 14–18 years old population in Finland. FINSEX 2007 is a population based sampling survey of 2590 adults, and the register-based marriage statistics includes all new marriages in Finland in 2008.

The new partner acquisition rate 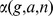 for *n = *0, i.e., for the first partner, was estimated from the age-specific proportions 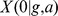 of those with no lifetime partners in the SHP study and the FINSEX 2007 data. For *n >*0, the estimation of *α* was based on the proportions 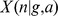 in the SHP study (teenagers) and on the annual partner number data in FINSEX 2007 (adults). We applied a likelihood function based on weighted squares of residuals and a prior with positivity and smoothness assumptions. The data are provided in the Tables S1–S2 in File S1.

The age-specific distribution of the partner age was taken to be a Beta distribution with the age-specific mean and variance estimated from the marriage statistics (Table S3 and Figure S4 in File S1). For teenagers, means and variances were extrapolated from the results for adults.

### Multiple hrHPV Type Infections

Infections with different hrHPV types were assumed to occur independently within a host. This corresponds to an assumption that any dependencies among type-specific infections at the population level are due to heterogeneity in the sexual behaviour and vaccination status. Specifically, in each 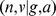 subpopulation, infections with different hrHPV types *j* (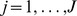) were considered independent, based on similar behavioural histories. Hence, the prevalence for all hrHPV infection in a 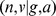 subpopulation at time *t* is
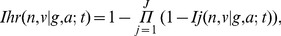
(2)where 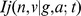 is the prevalence of type *j*. The prevalence of all hrHPV types in gender *g* at age *a* at time *t* is



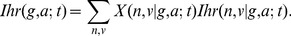
(3)By equations (2) and (3), it is enough to construct the transmission model separately for each hrHPV type in order to compute the all hrHPV prevalence.

### Single-type Transmission Model

**Figure 3:**
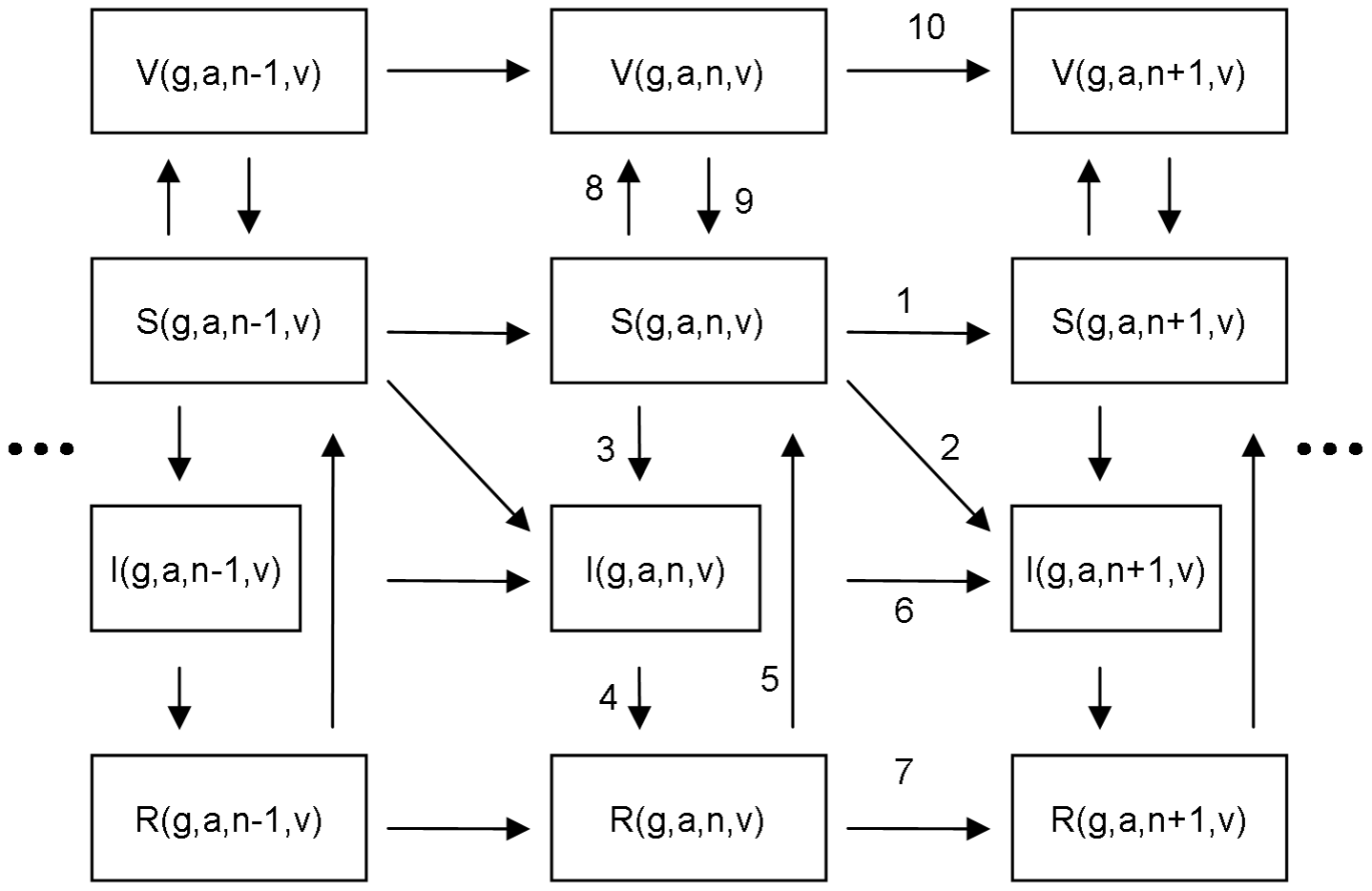
Transmission Model Structure for a Single HPV Type. The vertical flow corresponds to changes in the epidemiologic states susceptible (S), infectious (I), recovered (R), and vaccine-protected (V). The flow from left to right corresponds to an increasing lifetime partner number (*n*). The arrows describe possible transitions between different states: 1. Acquisition of a new partner without acquiring infection; 2. Acquisition of a new partner with acquiring infection (primary force of infection); 3. Acquisition of infection from the current partner (secondary force of infection, for *n >*0 only); 4. Clearance of infection; 5. Waning natural immunity; 6–7 and 10. Acquisition of a new partner for infected, recovered, and vaccine protected; 8. Take of vaccine protection; 9. Waning vaccine induced protection. The formulae for all transition rates are presented in File S1.

Figure 3 presents the structure of the transmission model for a single hrHPV type (for formulae, see Table S4 in File S1). The force of infection was divided into primary and secondary components, according to whether infection is acquired from a new partner or from the current one who has sex with someone else (secondary contact). The importance of the secondary force of infection was controlled with a weight parameter γ, which describes the intensity of individuals making secondary contacts.

The natural history of HPV infection was described with the infection-age (*τ*, time since infection) alone. In females, hrHPV types were assumed to clear with an infection-age dependent rate η(τ). The clearance of transient (new) infections was assumed to slow down type-specifically with τ, taking η(τ) to be a Weibull rate. We assumed that 5% of infections acquired more than 2 years ago become annually “old” with a common clearance rate (η*_pers_*) for all types (for implementation, see Figures S5–S6 in File S1). We call these “old” infections persistent infections in the following. In males, we assumed a constant type-specific clearance rate equalling the first year average of female rates [24]. After clearance, individuals were assumed to acquire natural immunity which wanes with rate *w*.

Vaccine-induced protection was modelled according to the “take” model. Among vaccinated individuals, vaccine efficacy thus determines the proportion moved from the susceptible state *S* to the completely vaccine-protected state *V* at the vaccination age (12 years). The duration of vaccine-induced protection was modelled through a waning immunity so that after an initial period of full protection for *T_vac_* years immunity subsequently wanes in an average of *μ_vac_* years. We assumed that HPV vaccination does not change the natural history of HPV infections, e.g., the clearance rates.

### HPV-related Parameters

For the transient (new) infections in females, the infection-age dependent hrHPV type-specific clearance rates were estimated from the Finnish unvaccinated control arm of the PATRICIA Phase III vaccine study [2]. The types were grouped to slow, moderate and fast clearance types (Table S5 and Figures S7–S8 in File S1).

**Figure 4:**
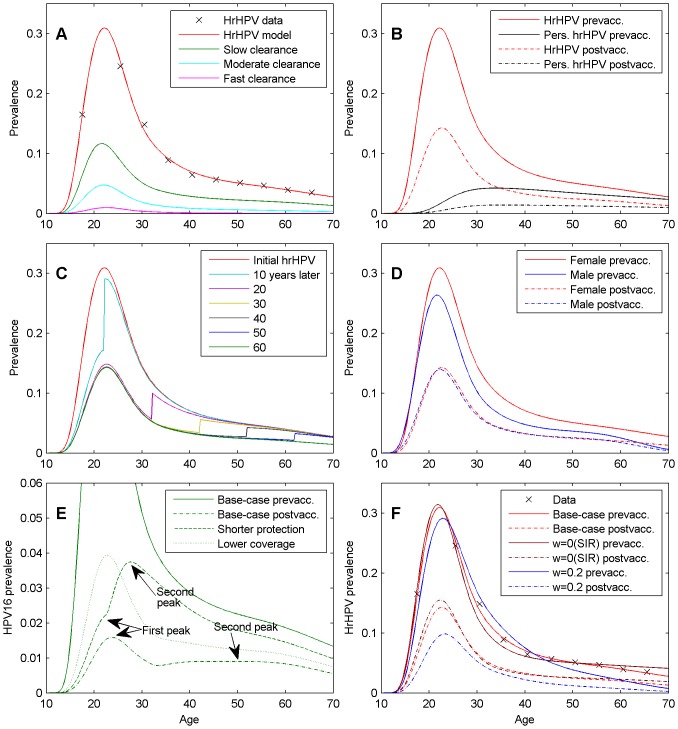
The Age-specific High-risk HPV (hrHPV) Prevalence in the Steady-state Before and After Vaccination. Unless otherwise stated, the results pertain to females under the base-case scenario. (a) The model prediction on the current hrHPV prevalence (upper curve) with the observed data (asterisks). The lower curves show the prevalence for three different single hrHPV types with low, moderate and fast clearance of infection (see Materials and Methods); (b) the prevalence of hrHPV and persistent hrHPV before and after vaccination; (c) hrHPV prevalence at different times since the onset of the vaccination program; (d) hrHPV prevalence in females and males, before and after vaccination; (e) HPV16 prevalence under different vaccine scenarios, waning vaccine protection induces a second peak in the prevalence curve; (f) model fits to hrHPV data under different waning rates of natural immunity (base-case, SIR, 0.2 1/year waning rate) and the corresponding post-vaccination prevalences.

The multiple-type transmission model was calibrated to the age-specific all hrHPV prevalence (Figure 4A, Table S6 in File S1) in the hrHPV screening trial [16]. These data include a population-based, non-type-specific, hrHPV prevalence among 75,000 women at screening ages 25, 30, ..., 65 in Finland in the prevaccination years 2004–2008. The prevalence data on teenagers came from the PATRICIA vaccine study. Three parameters were calibrated: the transmission probability β (probability to acquire HPV infection from a new infected partner), the clearance rate η*_pers_* of persistent infections, and the waning rate *w* of natural immunity. The model outcome (age-specific all hrHPV prevalence) was fitted to the data by sampling a weighted squares based posterior distribution. The weights were derived from the annual variation of age-specific hrHPV prevalence. During the calibration, the parameters of sexual behaviour, clearance of new infections, and the weight γ for the secondary force of infection were kept fixed.

### Effectiveness of Vaccination

The effectiveness of HPV vaccination was measured as a relative reduction in the prevalence of individual hrHPV types (for type-specific effectiveness) and all hrHPV types. The reductions were based on comparison of the pre- and post-vaccination steady states. The effectiveness was calculated among females 10–70 years of age and separately for the maximum age-specific prevalence.

Different vaccination characteristics were analysed as alternative scenarios. In the base-case (Table S7 in File S1), the vaccine efficacies against the vaccine and non-vaccine types (Table 1) were close to the best reported values (baseline HPV-naïve group in [4]). The vaccine and vaccination scenarios were [6]: higher/lower/no cross-protection, longer/shorter protection, and different vaccination strategies (Table 2).

**Table 1:** Model Predictions of the Steady-state High-risk HPV (hrHPV) Prevalence (prev.) Under the Base-case Vaccination Scenario.

		Pre-vaccination prevalence (%)		Post-vaccination prevalence (%)		Effectiveness of vaccination (%)	
hrHPV type	Vaccine efficacy (%)	Infection prev. (max)	Pers.inf. prev. (max)	Infection prev. (max)	Pers.inf. prev. (max)	Infection prev. (max)	Pers.inf. prev. (max)
all hrHPV	–	9.45 (30.93)	2.74 (4.24)	4.29 (14.28)	0.97 (1.42)	54.6 (53.8)	64.5 (66.6)
16	95	3.68 (11.66)	1.23 (1.23)	0.82 (1.59)	0.24 (0.36)	77.7 (86.4)	80.6 (80.8)
18	95	1.28 (4.75)	0.33 (0.52)	0.05 (0.11)	0.01 (0.02)	96.3 (97.8)	96.6 (96.8)
31	80	1.28 (4.75)	0.33 (0.52)	0.24 (0.70)	0.06 (0.08)	81.2 (85.2)	82.4 (83.7)
33	50	1.28 (4.75)	0.33 (0.52)	0.62 (2.13)	0.16 (0.24)	51.4 (55.3)	52.5 (53.9)
45	80	0.22 (0.99)	0.03 (0.04)	0.00 (0.00)	0.00 (0.00)	100 (100)	100 (100)
51	50	0.22 (0.99)	0.03 (0.04)	0.01 (0.03)	0.00 (0.00)	96.9 (97.3)	96.4 (96.8)
52	0	1.28 (4.75)	0.33 (0.52)	1.28 (4.75)	0.33 (0.52)	0 (0)	0 (0)
35, 39, 56, 58, 59, 66, 68	0	0.22 (0.99)	0.03 (0.04)	0.22 (0.99)	0.03 (0.04)	0 (0)	0 (0)
all hrHPV male	–	7.21 (26.38)	–	4.16 (13.96)	–.	42.3 (47.1)	–

Infection includes both transient and persistent infections (Pers.inf.). The prevalence refers to all females 10–70 years of age. The maximum prevalence (max) is the highest value of age-specific prevalence (cf. Figure 4). The effectiveness of HPV vaccination is defined as a relative reduction in the corresponding prevalence (post-vaccination vs pre-vaccination era). Base-case vaccination scenario: 80% vaccination coverage among women; wide range of type-specific protection against HPV16, HPV18, HPV31, HPV33, HPV45, and HPV51; 20 years duration of vaccine induced protection waning on average in the next 20 years.

**Table 2:** High-risk HPV (hrHPV) Prevalence and Effectiveness of HPV Vaccination Under Different Vaccination Scenarios.

Scenario	Coverage of vaccination (%)	Prevalence (%)			Effectiveness of vaccination (%)			
		hrHPV infection	hrHPV infection with onlydirect protection	hrHPV Pers.inf.	Direct	Herd	hrHPV infection	hrHPV Pers.inf.
Pre-vaccination	–	9.5	–	2.7	–	–	–	–
Base-case	G 80	4.3	5.9	1.0	37.8	16.8	54.6	64.5
Lower cross-protection	G 80	5.6	6.6	1.3	29.8	11.5	41.3	52.8
No cross-protection	G 80	6.1	7.0	1.5	25.9	9.4	35.4	46.7
Shorter protection, all types	G 80	6.1	7.1	1.5	24.5	10.5	35.0	44.1
Shorter protection, only non-vaccine types	G 80	4.8	6.2	1.1	34.0	14.9	48.9	59.5
Lower coverage	G 60	5.5	6.8	1.4	28.4	13.1	41.5	49.2
Lower cross-protection	G 80, B 40	5.1	6.6	1.1	29.8	16.2	46.0	58.2
Shorter protection, all types	G 80, B 40	5.6	7.1	1.4	24.5	15.9	40.3	50.4
Lower coverage	G 60, B 40	4.6	6.8	1.1	28.4	23.2	51.5	60.4
Higher coverage	G 90	3.8	5.4	0.8	42.6	17.7	60.2	70.9
Base-case with boys	G 80, B 40	3.7	5.9	0.8	37.8	23.4	61.3	71.5

The prevalence refers to hrHPV prevalence among females 10–70 years of age. Base-case vaccination scenario: 80% vaccination coverage among women; wide range of type-specific protection (Table 1); 20 years duration of vaccine induced protection waning on average in the next 20 years. Lower cross-protection: vaccine efficacy 50% against type 31, and 0% against other non-vaccine types; Shorter protection: 10 years duration of vaccine induced protection waning on average in the next 10 years; G/B the coverage of vaccination for girls/boys. High-risk HPV infection includes both transient and persistent infections (Pers.inf.). The effectiveness of HPV -vaccination is defined as a relative reduction in the corresponding prevalence (post-vaccination vs pre-vaccination era).

To study indirect protection (herd effect), the prevalence of infection was first determined without the transmission model assuming only direct protection: the prevalence of hrHPV types were kept unchanged in the non-vaccinees and set according to the vaccine efficacies in the vaccinees. The importance of indirect protection was then assessed with the given coverage of vaccination as the difference between the steady-state hrHPV prevalences under the two scenarios (the full model vs. only direct protection).

### Alternative Model Settings

The impacts of different model assumptions were investigated by comparison of different model settings (Table 3). The model was re-calibrated for each model setting. The weight γ for the secondary force of infection was assigned values from 0 to 0.8 (base-case γ = 0.4). The duration of natural immunity was varied from lifelong (SIR, susceptible-infected-recovered model) to very short (a model closer to SIS) by setting *w* = 0, and 0.2, respectively, and the remaining two calibration parameters were re-estimated. To study the sensitivity to the peak incidence of annual new partners, the annual new partner numbers at age 20–24 were increased by 20%.

**Table 3:** Alternative Model Settings.

Setting	Model fit				Post-vaccination prevalence (%)	
	WSR	η*_pers_* mean (SD)	β mean (SD)	*w* mean (SD)	hrHPV infection prev. (max)	hrHPV pers.inf. prev. (max)
Base-case	3.0	0.026 (0.005)	0.747 (0.012)	0.037 (0.006)	4.29 (14.28)	0.97 (1.42)
*w = *0 (SIR)	19.7	0.007 (0.002)	0.808 (0.012)	–	4.54 (15.49)	1.35 (1.82)
*w = *0.2 (∼SIS)	49.8	0.294 (0.048)	0.623 (0.006)	–	2.75 (9.84)	0.12 (0.36)
γ = 0	2.6	0.026 (0.004)	0.871 (0.017)	0.044 (0.005)	4.17 (13.74)	0.99 (1.45)
γ = 0.8	3.3	0.026 (0.005)	0.671 (0.010)	0.034 (0.006)	4.35 (14.48)	0.97 (1.40)
+20% partners	14.8	0.021 (0.005)	0.658 (0.014)	0.037 (0.008)	4.34 (16.28)	1.07 (1.55)
Male SIS	6.4	0.020 (0.003)	0.653 (0.008)	0.008 (0.001)	3.84 (12.46)	0.97 (1.37)

The model fit and outcomes (post-vaccination prevalence) under different model assumptions about duration of natural immunity (1*/w*), weight of the secondary force of infection (γ base-case* = *0.4), increased new partner acquisition rate for young adults (+20% partners), and different natural immunity waning model (SIS) only for males. The measure of model fit is a weighted sum of squared residuals (WSR). The calibrated model parameters: clearance rate of persistent infection (η*_pers_*), transmission probability per partnership (β), waning rate of natural immunity (*w*). The mean and standard deviation (SD) are given for each parameter. The high-risk HPV (hrHPV) infection includes both transient and persistent infections (pers.inf.).

The performance of each model setting was evaluated with a weighted sum of squared residuals (WSR), computed from the hrHPV prevalence. A small (large) WSR value means that the model fit is good (poor), i.e., the data agree (do not agree) with the model setting assumptions.

## Results

The transmission model reproduced accurately the observed population prevalence of hrHPV in Finland (Figure 4A). All three calibration parameters were identifiable in the base-case: the transmission probability was 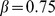 (with standard deviation SD = 0.01), 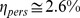 (SD 0.5%) of persistent infections cleared annually, and only 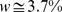 (SD 0.6%) of recovered individuals lost natural immunity per year. The estimated patterns of sexual contacts agreed well with the population-based data (Figure 2). According to the model, at the sexually most active ages about one fourth of women with hrHPV were infected with multiple types. The proportion decreased with age.

### Clearance of Transient (New) HPV Infections

Based on the cohort data [2], HPV16 formed slow, HPV18, HPV31, HPV33, and HPV52 moderate, and the other hrHPV types fast clearance groups. The clearance of infections slowed down with infection-age (i.e. the Weibull shape parameters were less than one, see Figure S8 in File S1). The mean (median) duration of infection for the slow, moderate, and fast clearance groups were 23(12), 12(6), and 7(4) months, and 4.4%, 2.0%, and 0.5% of these infections became eventually persistent, respectively.

### Effectiveness of HPV Vaccination

Unless otherwise stated, all results below apply to females between 10 and 70 years of age. Before vaccination, the prevalence (maximum prevalence) of hrHPV and of persistent hrHPV was 9.5% (30.9%) and 2.7% (4.2%), respectively (Table 1). Under the base-case vaccination scenario (80% vaccination coverage among women; wide range of type-specific protection against HPV16, HPV18, HPV31, HPV33, HPV45, and HPV51 (Table 1); 20 years duration of vaccine induced protection (*T_vac_*) waning on average in the next 20 years (*µ_vac_*)), the steady-state prevalence was 4.3% (14.3%), and 1.0% (1.4%), for hrHPV and persistent hrHPV, respectively (Table 1, Figure 4B). The cross-protection explained about 1/3 of the effectiveness of vaccination against hrHPV. Among individual HPV types, the absolute reduction in prevalence was highest for HPV16, but the relative effectiveness of vaccination was higher for the faster clearing HPV types (18, 45, and 51) than for the slowly clearing HPV16 (Table 1).

The proportion of persistent infections increased with age both in the pre- and postvaccination situations (Figure 4B). The effectiveness of vaccination against any individual hrHPV type depended on the clearance rate, but was similar against infection and persistent infection. However, the effectiveness was better against all persistent hrHPV infections (64.5%) as compared to all hrHPV infections (54.6%).

HPV vaccination separates the population into vaccinated and unvaccinated birth cohorts (Figure 4C). In practice, the unvaccinated birth cohorts do not benefit from vaccination. Even many decades after the start of vaccination program, only unvaccinated cohorts with ages close to the vaccinated ones experience a minor reduction in hrHPV prevalence. In contrast, the prevalence among the vaccinated birth cohorts is close to the eventual steady-state already after 10 years of starting the vaccinations and at the steady-state after 20 years.

In the new steady-state, among females indirect protection explained about 1/3 of the effectiveness of vaccination against hrHPV in the base-case scenario (Table 2). In males, the decrease in hrHPV prevalence was solely due to indirect protection. Among males between 10–70 years of age, the hrHPV prevalence was 7.2% before the vaccination program, and 4.2% in the post-vaccination steady state, corresponding to 42.3% effectiveness of vaccination (Figure 4D).

### Vaccination Scenarios

Table 2 summarises the sensitivity of model predictions to different vaccine and vaccination scenarios. Under each of the scenarios associated with weaker vaccine impacts (lower cross-protection with vaccine efficacy 50% against type 31, and 0 against other non-vaccine types; shorter *T_vac_* = *μ_vac_ = *10 years protection for all types; lower 60% coverage for girls), the effectiveness of vaccination was significantly worse than in the base-case. With shorter protective duration for the non-vaccine types the scenario was closer to the base-case than the corresponding shorter protective duration scenario for all types. A moderate coverage among girls was compensated by vaccinating also boys (girls 60%+boys 40%). However, this was not the case with weak vaccine-induced cross-protection or short protective duration for all types. Increasing the coverage among girls from 80% to 90% corresponded to the same effectiveness among females as vaccinating 80% girls and 40% of boys.

Waning of vaccine-induced protection was associated with a second increase in the prevalence of some HPV types in older women (e.g. Figure 4E for HPV16). A shorter duration of protection brought the increase earlier and more notable. The post-vaccination prevalence, however, remained smaller than the pre-vaccination prevalence for all ages.

### Alternative Model Settings

The model outcomes (Table 3, Figure 4) in the base-case analysis, in which the natural immunity waning rate was one of the calibrated parameters, were closer to those obtained when assuming life-long immunity (SIR) than very short durations of immunity (i.e. models close to SIS). In particular, the SIS model was not supported by data (large WSR). Interestingly, where the model strongly suggested a long-lasting natural immunity in women, the female hrHPV prevalence data were not informative about the duration of natural immunity in males. There was no significant difference in the model fit between SIS and SIR models applied only to males, but using SIS for males produced a better effectiveness of vaccination.

As the weight for the secondary force of infection was varied, the calibrated value for transmission probability changed contrariwise. Overall, however, the assumptions about the secondary force of infection had only little impact on the model fit or on the predicted effectiveness of vaccination as shown by the stable WSR (Table 3). Increasing the number of annual new partners in the most active adults (age 20–24) by 20% implied a decrease in the transmission probability but the other parameters and the effectiveness of vaccination remained unchanged. The model fit was, however, worse under this scenario (larger WSR, Table 3).

### Parameter Uncertainty

The influence of parameter uncertainty in model predictions was small (Table S8 and Figure S9 in File S1). There was much more variation in the model outcome across different vaccination scenarios and model settings.

## Discussion

We constructed a compartmental transmission model for single and multiple infections with 14 hrHPV types to predict the overall effectiveness of different vaccination strategies against hrHPV infection. The model reproduced adequately the current hrHPV prevalence in Finland. Assuming 80% vaccination coverage among girls, the model predicted approximately 55% reduction in the hrHPV prevalence and a higher 65% reduction in persistent hrHPV prevalence in females. For males, not vaccinated in the base-case, and females the model predicted 42% and 17% decreases in hrHPV prevalence, respectively, solely due to indirect protection (herd effect). The herd effect from vaccinating also males compensated a low coverage of vaccination among women.

Several lessons on the HPV natural history were learned by relating data on sexual behaviour to age-specific hrHPV prevalence through modelling. First, the considerably high hrHPV prevalence among older women can mainly be explained by persistent hrHPV infections. In particular, the level of sexual activity alone could not account for the slowly decreasing hrHPV prevalence in women of age 40 and over. Second, the rapid decrease in the prevalence after 25 years of age is due to both the decreasing sexual activity and acquired natural immunity. Third, to adequately describe the peak in the hrHPV prevalence in young women (ages 20–25), the transmission probability needs to be relatively high.

The presence of persistent hrHPV infections has important implications for the effectiveness of HPV vaccination, consistent with the critical role of the duration of infection in cervical carcinogenesis [25]. Our model assumed a prophylactic vaccine and therefore the effectiveness against infection and the effectiveness against persistent infection for any individual hrHPV type were at the same level. However, because the licensed vaccines include HPV16 and HPV18, which are slower clearing and thus more likely to become persistent, HPV vaccination appeared to have better effectiveness against all persistent hrHPV than against all hrHPV infection. This agrees with the observation of increasing vaccination effectiveness against increasingly severe cervical lesions [2], [3]. Our model with multiple types could thus explain the difference in the effectiveness against all hrHPV and all persistent hrHPV without the need of an additional mechanism, e.g. assuming that the vaccine would also prevent infection becoming persistent.

A high rate of waning immunity against hrHPV infection is unlikely as the model was not able to explain the rapid decrease in hrHPV prevalence after the peak prevalence under such a scenario. This does not rule out re-infections with the same type [26] as individuals may still lose their immunity. However, the dynamics of hrHPV appears to be closer to models with life-long immunity (SIR) than models without any immunity (SIS). With any given levels of vaccination coverage and vaccine efficacy, the SIR model sets a lower bound for the effectiveness of vaccination as the immunisation needs to replace the lifelong natural immunity with vaccine-induced protection. Nevertheless, the predicted overall effectiveness of HPV vaccination was significant.

Findings about a high transmission probability and a long duration of natural immunity are consistent with previous estimates, e.g., in [11], [14]. However, model simplifications such as not modelling condom use, smoking, and in our case also cervical cancer screening, were included implicitly in the calibration parameters. Note that similar issues apply also when corresponding parameters are estimated from trial data. For example, in a screening-based study [27], the clearance of hrHPV types was slower than what we estimated for a younger study population [2]. The difference can be explained with different proportions of persistent infections in the study populations. As a corollary, all parameters should be interpreted within the model context.

The limited herd effect in the older unvaccinated birth cohorts is due to the relatively narrow age-distribution of contacts. Moreover, during the first few years after the start of a vaccination program, when the vaccinated cohorts are still young, the direction of the HPV infection is mainly from older (unvaccinated) age cohorts to younger (vaccinated) ones, and the older age cohorts do not really benefit from the vaccination program.

Sexual networks [28] were modelled through a secondary force of infection. The estimates of the weight of secondary force of infection and the transmission probability (*β*) were coupled. If the secondary force of infection was given less (more) weight, then, the transmission probability was estimated higher (lower) so that the narrower (wider) paths of transmission were compensated. Nevertheless, this interplay had only a little influence on the predicted effectiveness of vaccination. The influence of a secondary force of infection might be higher with a different sexual behaviour pattern.

The clearance of hrHPV types was modelled to depend on infection-age. Our observation of the clearance slowing down with infection-age is consistent with a previous analysis, in which the infection-age dependent clearance rates were also modelled in a single HPV16 type mode [10]. Naturally, changing the definition of persistent infections would change the estimate of the clearance rate of persistent infections.

Interactions between different hrHPV types were modelled in the simplest way by assuming independence of types within a host (i.e., no natural infection/immunity derived cross-protection or within-host competition between different types). In addition, all three calibrated parameters were considered common to all hrHPV types. In our approach, variation in the hrHPV prevalence between types before vaccination was thus solely due to differing clearance rates. This was partly due to the fact that type-specific hrHPV prevalence data at the screening ages were not available. Nevertheless, the model was able to produce differences in the persistence between hrHPV types, in agreement with known differences in their oncogenicity, as well as a higher effectiveness of vaccination against all persistent hrHPV infection.

Limiting the model to infection without considering progression and screening simplified the analysis. It also makes our results generalisable to different countries, although they were based on data from Finland, as any differences in screening policies could be avoided. It should be noted that rapid changes, e.g., in HPV16 infection epidemics have occurred in Finland [29]. However, possible changes in sexual behaviour will have a much faster effect on hrHPV incidence than on the cancer incidence, and hence, the steady state assumption according to which the current hrHPV prevalence data correspond to the current sexual behaviour is at least partially justified.

Heterogeneity in sexual activity was modelled with an evolving lifetime partner number instead of predetermined sexual activity groups. Importantly, the lifetime partner number is a measurable variable, whereas the activity group is always a hyperparameter which cannot be observed directly. In our model, individuals with many lifetime partners correspond to high activity groups. We modelled HPV transmission in the heterosexual pair formation only. Relaxing the heterosexuality assumption might decrease the predicted herd effect [30] as the infection would have alternative paths to transmit.

The model predictions on the effectiveness of vaccination were not sensitive to different relevant model settings, in which the model fitted well to data. The only exception regarded the duration of natural immunity in males, for which the base-case was close to the lower bound for the effectiveness. Conversely, vaccination scenarios had much higher impact on the effectiveness. A suboptimal vaccine or vaccination program yielded remarkably worse effectiveness of vaccination. However, the low impact of a moderate (60%) vaccination coverage among girls could be compensated by the herd effect through vaccinating a reasonable proportion (40%) of boys.

There are some caveats in our analysis that should be highlighted. First, limiting the analysis to HPV infections and ignoring screening of cervical cancer, besides simplifying the analysis, is also a limitation, because some infections are treated after screening. Second, the model assumption of independent types means that any interactions between HPV types could not be addressed, including the possibility of cross-protection [31]. Third, it is possible that the vaccine-induced protection against some HPV types differ from those considered in our scenarios.

Our analysis has several implications. The ability of hrHPV to persist, the long natural immunity, and high transmissibility imply that both vaccination coverage and vaccine efficacy need to be high for elimination of infection. As a consequence of the considerable remaining prevalence of hrHPV infection, it seems necessary to continue cervical cancer screening in vaccinated populations, although possibly in new optimised forms. Especially, due to only a minor herd effect from vaccinated to unvaccinated cohorts, screening should be continued intensively among the unvaccinated cohorts. The increasing proportion of persistent infection with age should also be taken into account when developing screening programs. The analysis also suggests that monitoring hrHPV infection could be advantageous as part of cervical cancer screening.

In conclusion, vaccination of girls is expected to reduce the prevalence of all persistent hrHPV even more than the all hrHPV prevalence. If the coverage of vaccination among girls is low, it is more efficient, when female hrHPV prevalence is considered, to increase the coverage among girls than vaccinate boys. If this fails, however, it is possible to reduce the female hrHPV prevalence indirectly by vaccinating also boys. If the start of HPV vaccination program is postponed one can not rely on a herd effect to get protection afterwards for the unvaccinated, older bith cohorts. Finally, outcomes from the current transmission model can be applied as inputs to a disease progression model. Combinations of transmission and disease progression models are needed in optimising comprehensive HPV disease prevention programs.

## Supporting Information

File S1 **Detailed model specification and supportive figures including all data used as input in the analyses of this paper.**
(PDF)
