# Supplementary material for: Impact of Vaccination on 14 High-Risk HPV Type Infections: A Mathematical Modelling Approach
Source: PLoS One. 2013 Aug 29;8(8):e72088. doi: 10.1371/journal.pone.0072088 (PMC3756967; doi:10.1371/journal.pone.0072088)
Supplement: File S1 — Detailed model specification and supportive figures including all data used as input in the analyses of this paper. (PDF) [file pone.0072088.s001.pdf]

SUPPLEMENTARY MATERIAL

Impact of Vaccination on 14 High-Risk HPV type infections: A Mathematical Modelling Approach

Simopekka Vänskä, Kari Auranen, Tuija Leino, Heini Salo, Pekka Nieminen, Terhi Kilpi, Petri Tiihonen, Dan Apter, and Matti Lehtinen

Contents

Contact structure **2**

a Behavioural subpopulations 2

b Age-specific partner number distribution 2

c Estimation of new partner acquisition rates 2

d Partner distributions 7

Epidemiologic model **9**

a Model equations 9

b New (transient) infection clearance rates 12

c Model calibration 15

Contact structure

a. Behavioural subpopulations

Let $X(n, v|g, a; t)$ be the proportion of individuals with n lifetime partners and vaccination status v (v = vaccinated/unvaccinated) among those of gender g ($g = f/m$ = female/male) and age a at time t. Assume that the lifetime partner number in the (g, a) subpopulation does not depend on the vaccination status or the calendar time. Then the lifetime partner number distribution is

$$X(n|g, a, v; t) = X(n|g, a),$$

and

$$X(n, v|g, a; t) = X(n|g, a)X(v|g, a; t),$$

where $X(v|g, a; t)$ is the proportion of vaccinated in the subpopulation at time t.

b. Age-specific partner number distribution

In a steady state situation of sexual behaviour the new partner rates $\alpha = \alpha(g, a, n)$ are independent of time t. The lifetime partner number distribution $X(n|g, a)$, $n = 0, 1, 2, \ldots$, in a (g, a) subpopulation is then the solution of a continuous-time Markov process

$$\begin{cases} X'_{g,0}(a) &= -\alpha(g, a, 0)X_{g,0}(a), \\ X'_{g,n}(a) &= \alpha(g, a, n-1)X_{g,n-1}(a) - \alpha(g, a, n)X_{g,n}(a), \end{cases} \tag{1}$$

where $X_{g,n}(a) := X(n|g, a)$, and the initial state (at the model entry age $a_0 = 10$ years) is

$$\begin{cases} X_{g,0}(a_0) = 1, \\ X_{g,n}(a_0) = 0, \quad n > 0. \end{cases}$$

c. Estimation of new partner acquisition rates

For $n = 0$, the new partner acquisition rate $\alpha(g, a, 0)$ was specified in terms of three parameters (C_g, M_g, θ_g) by

$$\alpha(g, a, 0) = (C_g/c_g)(a - a0)^{k_g} \cdot \exp(-(a - a0)/\theta_g), \tag{2}$$

$$k_g = (M_g - a_0)/\theta_g, \quad c_g = (k_g\theta_g)^{k_g} exp(-k_g).$$

The parameters correspond to maximum rate (C_g), age of the maximum rate (M_g), and width of the decrease in the age (θ_g). The new partner acquisition rate (2) determines the function $X(0|g, a)$ in (1).

The new partner acquisition rate (2) was estimated from the age-specific proportions $X(0|g, a)$ in the SHP and FINSEX 2007 datasets [21,22] by applying a weighted squares of residuals based

posterior distribution (Figure S1, Table S1-S2). Females begin their sex life slightly earlier than males. For both males and females, the proportion of individuals with no lifetime partners remains above zero over all ages.

For $n > 0$, we parametrised

$$\alpha(g, a, n) = \phi_g(a) n^{p(a)}, \tag{3}$$

where ϕ_g represents the age dependency in the new partner acquisition rate. The exponent

$$p(a) = 1 - 0.9 \cdot \exp(-0.25 \cdot (a - 10))$$

for the partner number was set so that $p(a) \approx 1$ for adults and $p(a) \approx 0.7$ for teenagers. The linear dependence on n is natural for adults, because doubling the intensity of partner acquisition implicates a double number of partners in average. Based on the SHP data, the dependence on n in teenagers was found to be approximately $\sim n^{0.7}$. The function ϕ_g was parametrised by writing

$$\phi_g(a) = \frac{3}{a - a_0 + 0.1} + \psi_g(a)$$

and the correction term $\psi_g(a)$ was parametrised by making a piecewise linear representation for the second derivative ψ_g''. This approach was needed because the singularity of ϕ_g makes it difficult to estimate it directly. The new partner acquisition rates (2)-(3) determine the proportions $X(n|g, a)$ in (1) and the new partner acquisition rate $A_g(a)$ in a (g, a) subpopulation:

$$A_g(a) = \sum_n A(g, a, n), \tag{4}$$

where

$$A(g, a, n) = X(n|g, a)\alpha(g, a, n). \tag{5}$$

The new partner acquisition rate (3) was estimated from the proportions $X(n|g, a)$ of the SHP study and the age-specific annual new partner numbers[1] adapted from the FINSEX 2007 study (Figure 3 of the main text, Figure S2-S3, Table S1-S2). The function $A_g(a)$ corresponds to the age-specific annual new partner numbers. We applied a likelihood function based on a weighted squares of residuals and a prior with positivity and constraint on $|A_g''(a)|$ and $-A_g'(a)$ for $a > 25$ (to avoid oscillation and over-fitting). The weight at age group j (with N_j responders and average annual new partner number A_j) was the inverse of the variance A_j/N_j, which was obtained by assuming new partners to accumulate as a Poisson process.

[1]Because the FINSEX 2007 data contained information about annual partner numbers, we interpreted the new annual partner numbers to be one less than the reported mean, i.e., we approximated that the first annual partner was not new but an old one from the previous year.

Table S1. The lifetime partner number by age in teenagers. The data are adapted from the School Health Promotion study [21]. N = number of responders, $p(n)$ = the proportion (%) having n lifetime partners. For age below 16, the proportions are from the primary school data. For age 16 and over, the proportion is a combination of high school and vocational school proportions, weighting the high school proportion with the percentige of girls/boys (60%/42%) starting high school in Finland.

	Male						Female					
Age	N	$p(0)$	p(1)	p(2)	p(3-4)	p(5+)	N	$p(0)$	p(1)	p(2)	p(3-4)	p(5+)
14.5	10665	87.3	6.3	2.3	1.5	2.6	11606	87.9	6.9	2.5	1.6	1.0
15.0	12297	84.2	8.1	2.8	2.0	2.8	12789	84.6	8.8	3.1	2.3	1.2
15.5	12496	77.1	11.7	4.3	3.1	3.8	12371	73.9	13.4	5.7	4.5	2.5
16.0	12951	71.9	14.0	5.8	3.9	4.5	12719	68.2	16.2	6.8	5.4	3.5
16.5	9287	62.4	17.3	8.4	6.8	5.1	10181	55.2	20.3	9.5	8.6	6.4
17.0	10161	57.4	19.5	8.9	7.8	6.4	10812	50.2	22.4	10.2	9.7	7.5
17.5	9493	51.2	20.9	10.3	9.5	8.1	9826	41.7	24.1	11.3	12.0	10.9
18.0	9349	44.4	22.1	12.3	11.0	10.3	9663	36.7	25.0	12.4	13.3	12.6

Table S2. The lifetime partner number by age in adults. The data are adapted from the FINSEX 2007 study [22]. N = the number of responders, $p(0)$ = the proportion (%) with no lifetime partners, A is the average annual new partner number.

	Male			Female		
Age	N	$p(0)$	A	N	$p(0)$	A
20-24	80	19	1.2	146	7	1.3
25-29	93	10	0.8	129	6	0.5
30-34	88	4	0.7	147	2	0.3
35-39	98	2	0.7	132	1	0.5
40-44	82	5	0.4	116	0	0.2
45-49	96	1	0.5	135	2	0.3
50-54	99	3	1.0	162	1	0.2
55-59	124	1	0.6	149	0	0.1
60-64	117	1	0.5	123	0	0.1
65-69	79	1	0.1	105	4	0.1

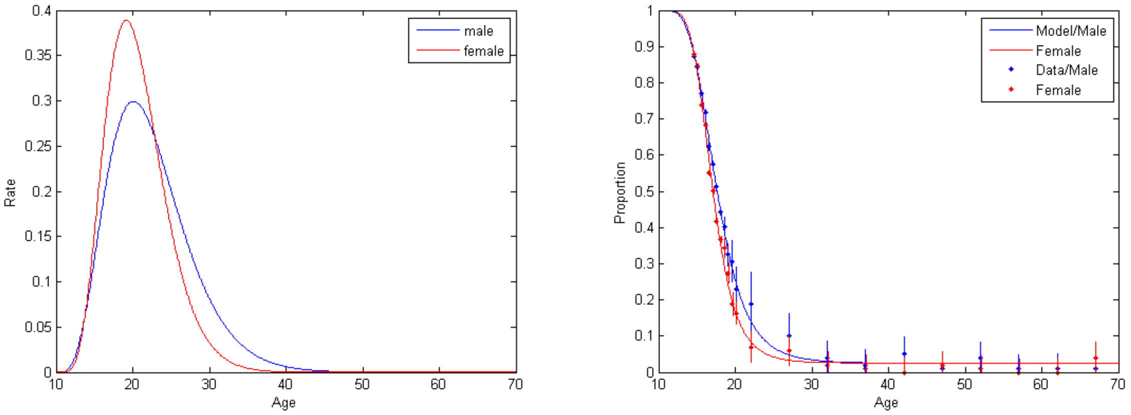

Figure S1. The new partner acquisition rates $\alpha(g, a, n)$ in those with no previous partners (i.e. when $n = 0$). This figure is supplementary to Figure 2 in the main text. Left: Estimated age-specific new partner acquisition rate $\alpha(g, a, 0)$ [1/year] (g =male/female blue/red). Right: Age-specific proportion of individuals having no lifetime partners ($X_{g,0}(a)$), model fit. Data points are equipped with the intervals describing the standard deviation.

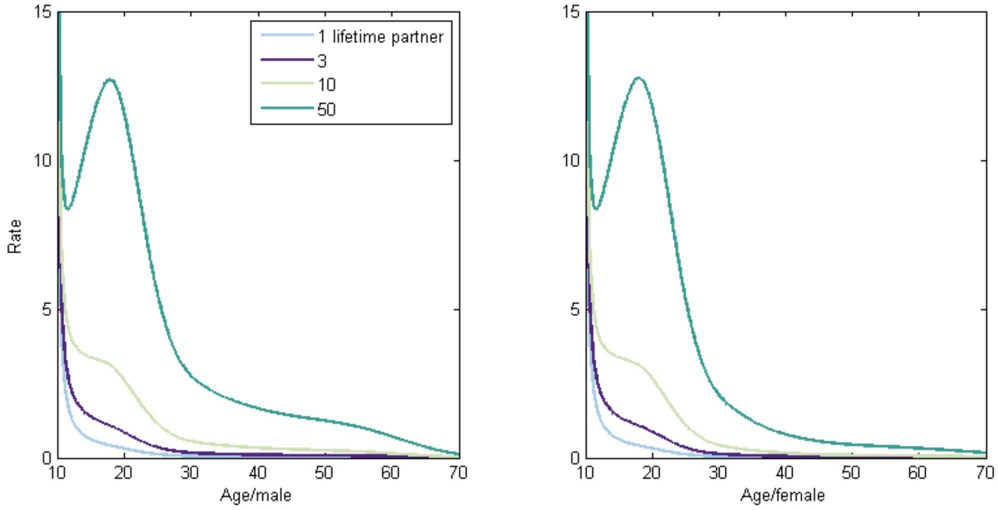

Figure S2. Age-specific new partner acquisition rates $\alpha(g, a, n)$ [1/year] with partner numbers $n = 1, 3, 10, 50$ (g =male/female left/right).

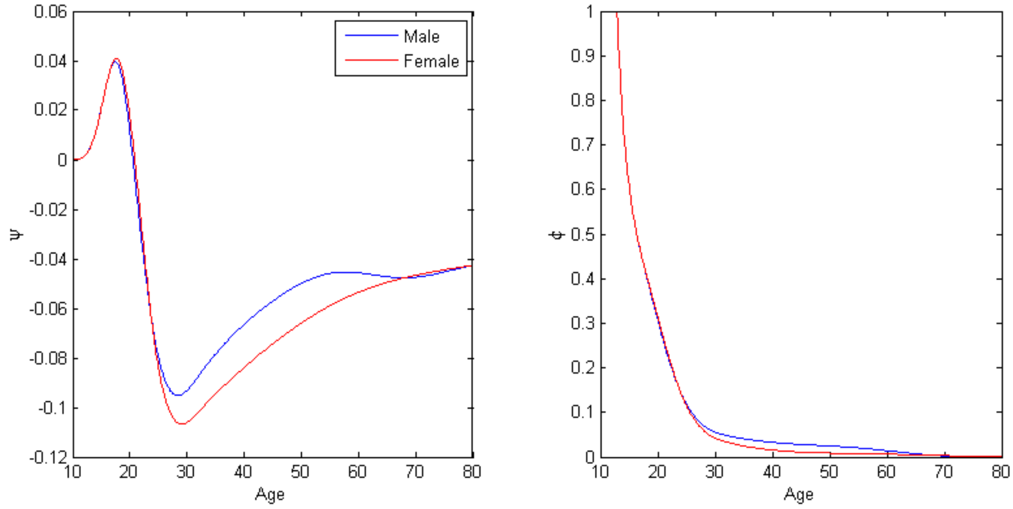

Figure S3. Function ϕ_g. The function $\phi_g = \phi_g(a)$ [1/year] was the age a dependent factor of the new partner acquisition rate $\alpha(g, a, n)$ for $n > 0$, see text (g =male/female blue/red). Left: Correction term ψ_g. Right: Function ϕ_g.

Table S3. The partner age difference (years) by age. The data were from the official statistics of marriage in Finland [23]. N = number of cases, Mean = the mean age difference, and SD = the standard deviation of age difference.

	Male (female partners)			Female (male partners)		
Age	N	Mean	SD	N	Mean	SD
-19	246	2.3	4.2	700	5.3	4.8
20-24	2871	0.8	4.2	4608	4.0	4.7
25-29	8001	-0.5	4.1	9256	2.5	4.5
30-34	7086	-2.2	4.7	5938	1.8	5.4
35-39	4033	-3.1	5.9	3285	1.7	6.2
40-44	2970	-3.4	6.6	2497	1.4	6.8
45-49	2490	-3.5	6.6	2334	0.8	6.8
50-54	1400	-4.5	6.9	1127	0.4	6.9
55-59	1000	-5.5	7.0	706	-0.4	7.0
60-64	550	-6.7	7.3	331	-1.5	7.2
65-69	194	-7.2	7.2	140	-2.1	6.5
70-	173	-6.8	7.8	92	-2.1	4.2

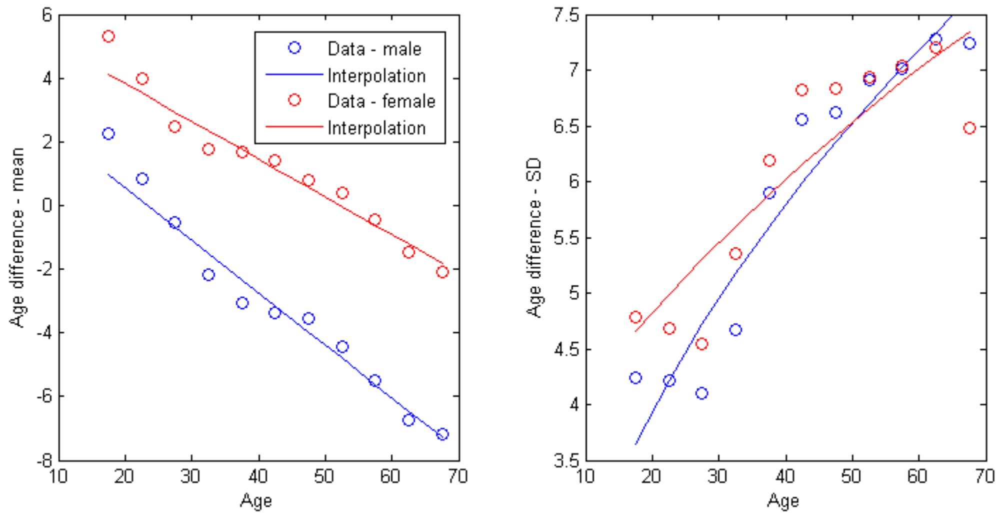

Figure S4. The estimated partner age difference (years) by age. Data points correspond to those in Table S3. Left/Right: Estimated mean/standard deviation (male/female blue/red).

d. Partner distributions

The disribution of contacts was constructed according to the age distribution of heterosexual pair formation, and the proportional mixing principle applied to partner number and vaccination status. Hence, the proportion of contacts in (g', a', n', v') that an individual in the (g, a) subpopulation make at time t is

$$\rho(g', a', n', v'|g, a; t) = \rho(a'|a, g)X(v'|g', a'; t)A(n'|g', a'), \tag{6}$$

for the opposite gender $g' = -g$. Here $\rho(a'|a, g)$ is the probability for an (g, a) individual that the partner is of age a' of opposite sex, $X(v'|a', g')$ is the probability for the vaccination status v', and

$$A(n'|g', a') = \frac{A(g', a', n')}{A_{g'}(a')},$$

see equations (4)-(5), is the probability to choose partner with n' lifetime partners among all in the (g', a') subpopulation by the proportional mixing principle.

The probability $\rho(a'|a, g)$ was taken to follow the $Beta(a_0, 100)$ distribution, with the mean and variance obtained from the official marriage statistics in Finland [23] (Figure S4, Table S3). For teenagers, the means and variances were linearly extrapolated from the results for adults. Both for males and females, the mean partner age difference was found to be a decreasing function of age, and negative for ages over 60. This can be explained by the larger supply of partners from younger age groups. The variance increased by age. Using the marriage data as the partner age distribution can be interpreted as taking the processes of (decision of) getting married and taking a new partner mutually independent.

Because all interaction between a and a' in (6) acts through $\rho(a'|a, g)$, the transmission model can be built based on the proportional subpopulations $X(n, v|g, a; t)$ without taking the mortality into account explicitly. When needed, the actual population size can be obtained by weighting the proportion with the corresponding population size.

Non-matching partner numbers between males and females in survey data is one problem that has to be handled. We did not force the overall partner numbers to match. This can be interpreted there being an extra-population out of the model population to which males are in contact, and epidemic states in this extra-population are distributed similarly as in the model population.

Epidemiologic model

a. Model equations

Figure S5 (copied here from the main text for convenience) presents the model structure for a single HPV type transmission. The transition rates are given in the Table S4.

The ageing of infection was modelled in terms of progression between five "infection-age" states (see Figure S6). The first four infection-age states I_i, $i = 1, \ldots, 4$, denote new (transient) infections. In each of these four states, an infected individual can either clear the infection (with a rate η_i depending on the HPV type and the infection-age state) or progress to the subsequent infection-age state with rate π_i ($\pi_1 = \pi_2 = \pi_3 = 2$/year). From the fourth state, the individual's infection may become "old" with rate $\pi_4 = 1/20$ per year. The individual may clear even the old infection with a clearance rate η_{pers}. This rate was assumed to be common to across all hrHPV types and it was estimated as part of the model calibration. It was then found that the rate was indeed much smaller than the (type-specific) rates initially after the infection. Accordingly, we opted to call the 'old infections' as persistent.

Table S4. Transition rates. Numbers corresponds to arrow numbers in Figure S5.

	Description	Symbol/Formula
1	Acquisition of new partner without acquiring infection	$\alpha(g,a,n)(1-\Lambda(g,a;t))$
2	Acquisition of new partner with acquiring infection (primary force of infection)	$\lambda_1(g,a,n;t) = \alpha(g,a,n)\Lambda(g,a;t)$
3	Acquisition of infection from current partner (secondary force of infection, for $n > 0$ only)	$\lambda_2(g,a,n;t) = \gamma\beta \sum_{n',v'} \lambda_1(-g,a,n')S(-g,a,n',v';t)$
4	Clearance of infection	$\eta(\tau)$
5	Waning natural immunity	w
6,7,10	Acquisition of new partner for infected, recoverd, and vaccine protected	$\alpha(g,a,n)$
8	Take of vaccine protection	$r_{vac}(g,a;t)$
9	Waning vaccine induced protection	$w_{vac}(g,a;t)$
	Notation	
	The probability for a susceptible in the (g,a) subpopulation to acquire infection from a new partner	$\Lambda(g,a;t) = \beta \sum_{n',v'} \int \rho(-g,a',n',v'\|g,a;t) \frac{I(-g,a',n',v';t)}{X(-g,a',n',v';t)} da'$
	HPV transmission probability (from a new infected partner)	β
	Weight for the secondary force of infection	γ

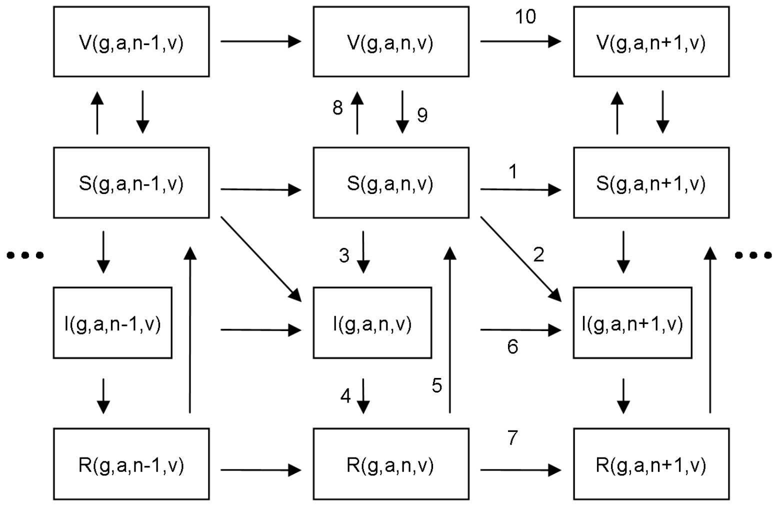

Figure S5. Transmission model structure for a single hrHPV type. The vertical flow corresponds to changes in the epidemiologic states susceptible (S), infectious (I), recovered (R), and vaccine-protected (V). The flow from left to right corresponds to increasing partner number (n). The transition rates are listed in Table S4.

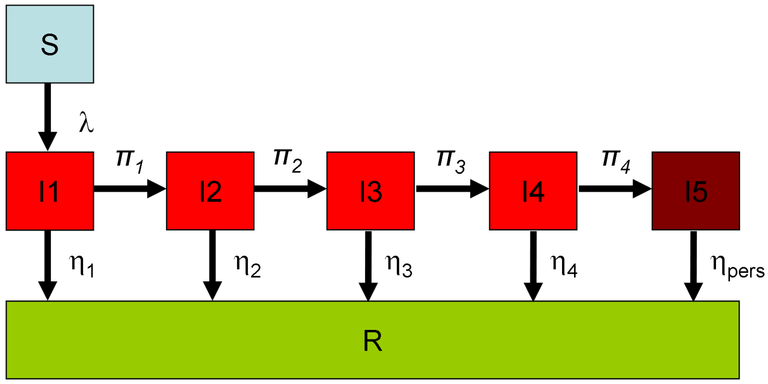

Figure S6. Ageing of hrHPV infection. A succeptible individual (S) acquires infection with rate λ and moves to infection-age state I_1. In each of the infection-age states I_i, $i = 1, \ldots, 4$, an infected individual can either clear the infection (with a rate η_i depending on the hrHPV type and the corresponding infection-age state) or progress to the subsequent infection-age state with rate π_i. The infections in the infection-age state $I_5 = I_{pers}$ are called persistent, and they clear with a rate η_{pers}, common to all hrHPV types.

The single-type transmission model is described in terms of the following system of differential equations:

$$
\begin{aligned}
&(\partial_t + \partial_a)S(g,a,n,v;t) = \alpha(g,a,n-1)(1-\Lambda(g,a;t))S(g,a,n-1,v;t)\\
&\quad + wR(g,a,n,v;t) + w_{vac}(g,a;t)V(g,a,n,v;t)\\
&\quad - (\alpha(g,a,n) + \lambda_2(g,a;t) + r_{vac}(g,a,v;t))S(g,a,n,v;t)\\
&(\partial_t + \partial_a)I_1(g,a,n,v;t) = \alpha(g,a,n-1)I_1(g,a,n-1,v;t)\\
&\quad + \lambda_1(g,a,n-1;t)S(g,a,n-1,v;t) + \lambda_2(g,a;t)S(g,a,n,v;t)\\
&\quad - (\alpha(g,a,n) + \eta_1 + \pi_1)I_1(g,a,n,v;t)\\
&(\partial_t + \partial_a)I_i(g,a,n,v;t) = \alpha(g,a,n-1)I_i(g,a,n-1,v;t) \qquad (i=2,3,4)\\
&\quad + \pi_{i-1}I_{i-1}(g,a,n,v;t)\\
&\quad - (\alpha(g,a,n) + \eta_i + \pi_i)I_i(g,a,n,v;t)\\
&(\partial_t + \partial_a)I_{pers}(g,a,n,v;t) = \alpha(g,a,n-1)I_{pers}(g,a,n-1,v;t) + \pi_4 I_4(g,a,n,v;t)\\
&\quad - (\alpha(g,a,n) + \eta_{pers})I_{pers}(g,a,n,v;t)\\
&(\partial_t + \partial_a)R(g,a,n,v;t) = \alpha(g,a,n-1)R(g,a,n-1,v;t)\\
&\quad + \sum_{i=1}^{4}\eta_i I_i(g,a,n,v;t) + \eta_{pers}I_{pers}(g,a,n,v;t)\\
&\quad - (\alpha(g,a,n) + w)R(g,a,n,v;t)\\
&(\partial_t + \partial_a)V(g,a,n,v;t) = \alpha(g,a,n-1)V(g,a,n-1,v;t)\\
&\quad + r_{vac}(g,a,v;t)S(g,a,n,v;t) - (w_{vac}(g,a;t) + \alpha(g,a,n))V(g,a,n,v;t).
\end{aligned}
$$

The vaccination rate $r_{vac}(g,a,v;t)$ is supported in the single age of vaccination ($a = 12$ years) so that the proportion, which is determined by the vaccine efficacy, is moved to vaccine protected state (only for $v =$ vaccinated).

The interpretation for the secondary force of infection formula is the following: Consider an individual in the (g,a,n) subpopulation (with a partner). Assume first that the partner of opposite sex $g' = -g$ belongs to a subpopulation $(-g,a',n',v')$. The partner takes new partners (has sex with someone else, secondary contacts) with a reduced rate $\gamma\alpha(-g,a',n')$. The reduction, determined by factor γ, is compared to the general rate $\alpha(-g,a',n')$ of such individuals. Because only susceptible can become infected, the rate at which the partner becomes infected is

$$
\begin{aligned}
&\gamma\alpha(-g,a',n')\Lambda(-g,a';t)S(-g,a',n',v';t)/X(-g,a',n',v';t)\\
&\qquad\qquad = \gamma\lambda_1(-g,a',n')S(-g,a',n',v';t)/X(-g,a',n',v';t).
\end{aligned}
$$

By summing over n', v', and by integrating over a' (and approximating the integral with the one

point approximation $a' = a$), we get that any partner becomes infected with rate

$$\sum_{n',v'} X(-g,a,n',v';t)\gamma\lambda_1(-g,a,n')S(-g,a,n',v';t)/X(-g,a,n',v';t)$$

$$= \sum_{n',v'} \gamma\lambda_1(-g,a,n')S(-g,a,n',v';t).$$

The transmission probability β is the probability to acquire HPV infection from a new infected partner. Now, we interpret that the existing partner, who becomes infected, is a new *infected* partner for the original individual. Hence, the original individual acquires infection with rate (the secondary force of infection)

$$\lambda_2(g,a,n;t) = \beta \sum_{n',v'} \gamma\lambda_1(-g,a,n')S(-g,a,n',v';t).$$

The prevalence of multiple-type infections is obtained from the equations (2)-(3) of main text. A similar approach can be used to compute the prevalence of any combination of hrHPV types.

b. New (transient) infection clearance rates

For the new infections, the hrHPV type-specific clearance rates were estimated from the cohort data in the control arm of PATRICIA phase III HPV vaccine study in Finland [2]. The data consists of type-specific frequences of incident infections (N) and infections at two/three consequtive test visits (N6/N12). Because the test visits were 6 months apart, we interpreted the frequences $N6/N12$ to correspond to the infections of (at least) 6/12 months infection-age. We adjusted the frequences to correspond to the limited 48 month follow-up time, Table S5.

We assumed that the clearance follows a Weibull distribution $F(\cdot; L, p)$ with scale L and shape p so that the clearance rate is the Weibull hazard

$$\eta(\tau) = \eta(\tau; p, L) = \frac{p}{L}\left(\frac{\tau}{L}\right)^{p-1}$$

for the infection of age τ. Let

$$q_{t,t'} = F(t'; L, p) - F(t; L, p)$$

be the probability that infection clears at the infection-age interval $[t, t']$ (years). Then

$$P((N, N6, N12)|L, p) = q_{0,0.5}^{N-N6} q_{0.5,1.0}^{N6-N12} q_{1.0,\infty}^{N12}.$$

First, the posterior distributions were computed for each type (Figure S7). Based on these distributions, we grouped hrHPV types to slow, moderate, and fast clearance types. HPV type 16 was the slowest clearance type. The moderate clerance group of hrHPV types was formed by

asking the rate $1/L$ to be below the average. The rest of hrHPV types formed the fast clearance group. Data were about α_7 and α_9 species HPV types. Other hrHPV types were assumed to be of fast clearance. Second, the posterior distributions were computed for each clearance group (Figure S8).

Finally, in each infection-age state I_i, $i = 1, \ldots, 4$, we set the clearance rate $\eta_i = \eta(\tau_i)$ by choosing an appropriate infection-age τ_i. We used $\tau_{1/2/3/4} = 0.25/0.75/1.25/1.75$ years.

Table S5. Type-specific durations of hrHPV infections in the control arm of PATRICIA phase III HPV vaccine study in Finland [2]. Left: N = Findings at the baseline plus new findings during the follow-up. $N6/N12$ = Infections at two/three consequtive test visits (6 months apart). Right: The corresponding data (N, $N6$, $N12$) adjusted for the 48 month follow-up time.

HPV	Non-adjusted			Adjusted		
Type	N	N6	N12	N	N6	N12
16	118+530	393	259	515.5	343.9	259.0
18	52+396	195	104	349.0	170.6	104.0
31	52+388	196	116	343.0	171.5	116.0
33	36+256	127	70	228.0	111.1	70.0
35	7+107	34	11	87.3	29.8	11.0
39	30+259	118	54	224.3	103.3	54.0
45	19+149	62	20	130.8	54.3	20.0
51	96+556			513.0		
52	55+480	251	137	415.0	219.6	137.0
56	46+341			301.8		
58	17+179	76	33	151.3	66.5	33.0
59	17+187	32	2	157.3	28.0	2.0
66	46+294			266.5		
68	31+369	123	52	307.8	107.6	52.0

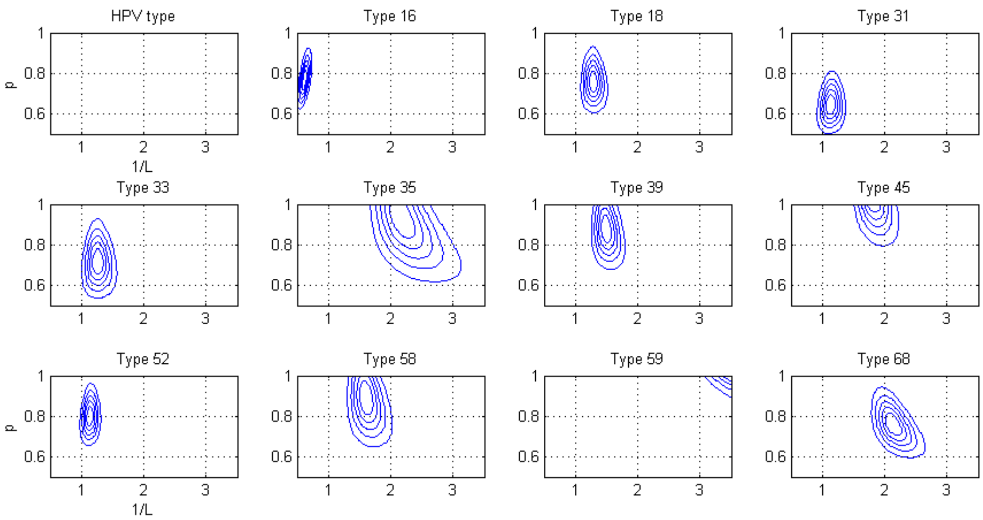

Figure S7. Posterior distributions of the clearance rate parameters. For each hrHPV type, the posterior distribution is for the two parameters (rate $1/L$ and shape p) of the Weibull clearance rate (hazard).

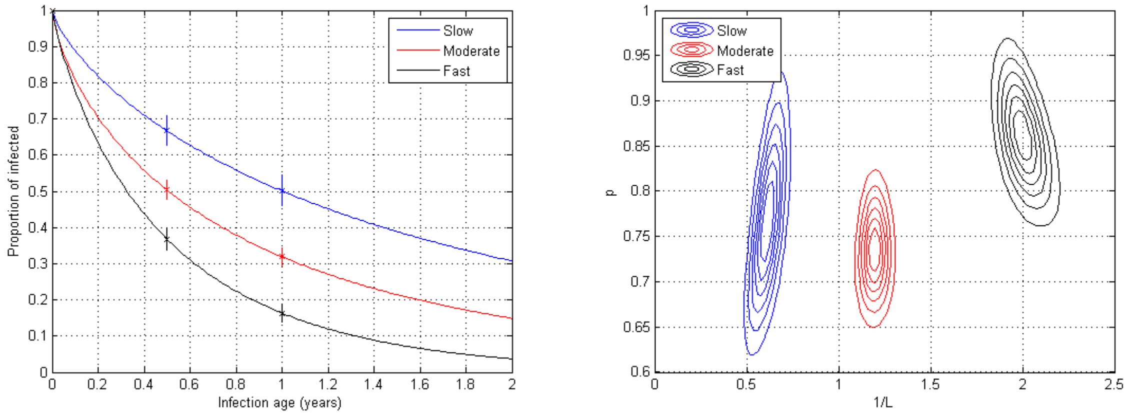

Figure S8. Infection-age dependent clearance by clearance group. Left: The survival functions of hrHPV in infection-age for slow (type 16), moderate (18, 31, 33, 52) and fast (the rest) clearance types. Right: Posterior distribution of Weibull shape (p) and rate ($1/L$). All three likelihoods are supported at $p < 1$, i.e., the clearance rates decrease by the infection-age.

Table S6. HrHPV prevalence data for women. HrHPV = age-specific average prevalence of hrHPV infection of years 2004-2008. N = number of participants (2004-2008) at the age. Age 17 is from the control arm of PATRICIA phase III HPV vaccine study in Finland [2]. Ages 25-65 are from the hrHPV screening trial [20].

Age	17	25	30	35	40	45	50	55	60	65
hrHPV prevalence	0.165	0.246	0.148	0.089	0.064	0.057	0.051	0.047	0.040	0.035
N	2399	4400	8174	7667	9401	9235	9223	9721	9830	3289

c. Model calibration

The model calibration involved the estimation of three parameters (transmission probability β, clearance rate of persistent infections η_{pers}, waning rate of natural immunity w). The model was calibrated to the population based age-specific all hrHPV prevalence screening study data [20], Table S6. Note that the prevalence data consist of prevalences x_i at the screening ages i rather than averages over age groups because the data were collected at the screening ages (5-yearly starting from 25).

The calibration was based on minimizing the weighted squares of residuals (the differences between model outcomes and the annual mean prevalence). In particular, we sampled the posterior of the three parameters with a Metropolis-Hastings algorithm applied to a weighted sum of squared residuals (WSR) based posterior distribution, i.e., the log-likelihood function was

$$\log P(x|\eta_{pers}, \beta, w)) = -\sum_i w_i (y_i - x_i)^2,$$

where the weights w_i correspond to the levels of "error" that were allowed between the model outcome $y_i = y_i(\eta_{pers}, \beta, w)$ and the data $x = (x_i)$ at the screening ages i (Figure 4A in the main text, Figure 8). For given model parameters, the age-specific epidemiologic state $y_i = y_i(\eta_{pers}, \beta, w)$ was computed as an endemic state by running the model for 100 years starting with any reasonable state (a state with some prevalence in sexually active population for each hrHPV type). As the number of individuals was very large in the screening study, the statistical uncertainty around the mean age-specific hrHPV prevalence for the whole study period (2004-2008) becomes very small when compared to the annual variation around that mean. To prevent too rigid a model fit, we used the annual variation (unbiased sample variance of annual prevalences $\approx 0.008^2$) in the hrHPV prevalence to derive weights w_i used in the weighted-squares based posterior distribution. The candidate sample point in the Metropolis-Hastings algorithm was drawn from a three-dimensional Gaussian distribution.

Table S7. List of model parameters (base-case).

Population parameters		Value	Source
Sexual debut			
	$C_f; C_m$ [1/year]	0.39; 0.30	Estimated
	$M_f; M_m$ [year]	19.1; 20.1	from
	$\theta_f; \theta_m$ [year]	1.5; 2.3	the studies
Accumulating partners			[21,22].
	$\phi_f(a), \phi_m(a)$	Fig. S3	
Partners ages			
	$\rho(a'\|f, a), \rho(a'\|m, a)$	Fig. S4	[23]
**HPV parameters**			
HPV-type and duration dependent clearance rates, Weibull parameters			
HPV type 16	(1/L,p)	(0.6,0.77)	Estimated from
18,31,33,52		(1.2,0.73)	[2]
Other hrHPV		(2.0,0.86)	
Natural immunity waning rate	w	0.037	Model fit to [20]
Weight for λ_2	γ	0.4	Base-case scenario
Transmission probability	β	0.747	Model fit to [20]
Clearance rate for persistent infection [1/year]	η_{pers}	0.026	Model fit to [20]
**Vaccination parameters**			
Coverage [%] for girls/boys		80/0	Base-case scenario
Duration for the protection [years]	(T_{vac}, μ_{vac})	(20,20)	Base-case scenario
Vaccine efficacy (%) by hrHPV type	Type 16, 18	95	Base-case scenario
	31, 45	80	Base-case scenario
	33, 51	50	Base-case scenario
	35,39,52,56,58,59,66,68	0	Base-case scenario
**Technical parameters**			
Progression from I_i, $i = 1, 2, 3$ [1/year]	π_i	2	Model choice
Progression from I_4 to persistent infections [1/year]	π_4	1/20	Model choice
Infection-age in I_i, $i = 1, ..., 4$	τ_i	0.25/0.75/1.25/1.75	Model choice
Model entering age [years]	a_0	10	Model choice
Maximum number of partners		200	Model choice

Table S8. Uncertainty in the predictions due to parameter uncertainty. This table corresponds to Table 1 of the main text and includes the standard deviations (SD) of corresponding cells. The standard deviations were computed by sampling the model parameters from the posterior distribution (see Figure S9).

	Pre-vaccination prevalence (SD%)		Post-vaccination prevalence (SD%)		Effectiveness vaccination (SD%)	
hrHPV type	Infection prev.(max)	Pers.inf. prev.(max)	Infection prev.(max)	Pers.inf. prev.(max)	Infection prev.(max)	Pers.inf. prev.(max)
all hrHPV	0.19(0.48)	0.19(0.15)	0.13(0.45)	0.07(0.07)	0.87(0.78)	0.65(0.65)
16	0.09(0.12)	0.08(0.06)	0.02(0.04)	0.02(0.03)	0.38(0.22)	0.32(1.04)
18	0.03(0.07)	0.02(0.02)	0.01(0.03)	0.00(0.00)	0.92(0.56)	0.75(0.77)
31	0.03(0.07)	0.02(0.02)	0.01(0.04)	0.01(0.01)	0.89(0.64)	0.79(0.84)
33	0.03(0.07)	0.02(0.02)	0.02(0.05)	0.01(0.01)	0.60(0.47)	0.54(0.58)
45	0.01(0.05)	0.00(0.00)	0.00(0.00)	0.00(0.00)	0.00(0.00)	0.00(0.00)
51	0.01(0.05)	0.00(0.00)	0.01(0.02)	0.00(0.00)	2.42(2.19)	2.25(2.19)
52	0.03(0.07)	0.02(0.02)	0.03(0.07)	0.02(0.02)	0(0)	0(0)
35,39,56,58, 59,66,68	0.01(0.05)	0.00(0.00)	0.01(0.05)	0.00(0.00)	0(0)	0(0)
all hrHPV male	0.20(0.41)	-	0.12(0.43)	-	1.37(0.91)	-

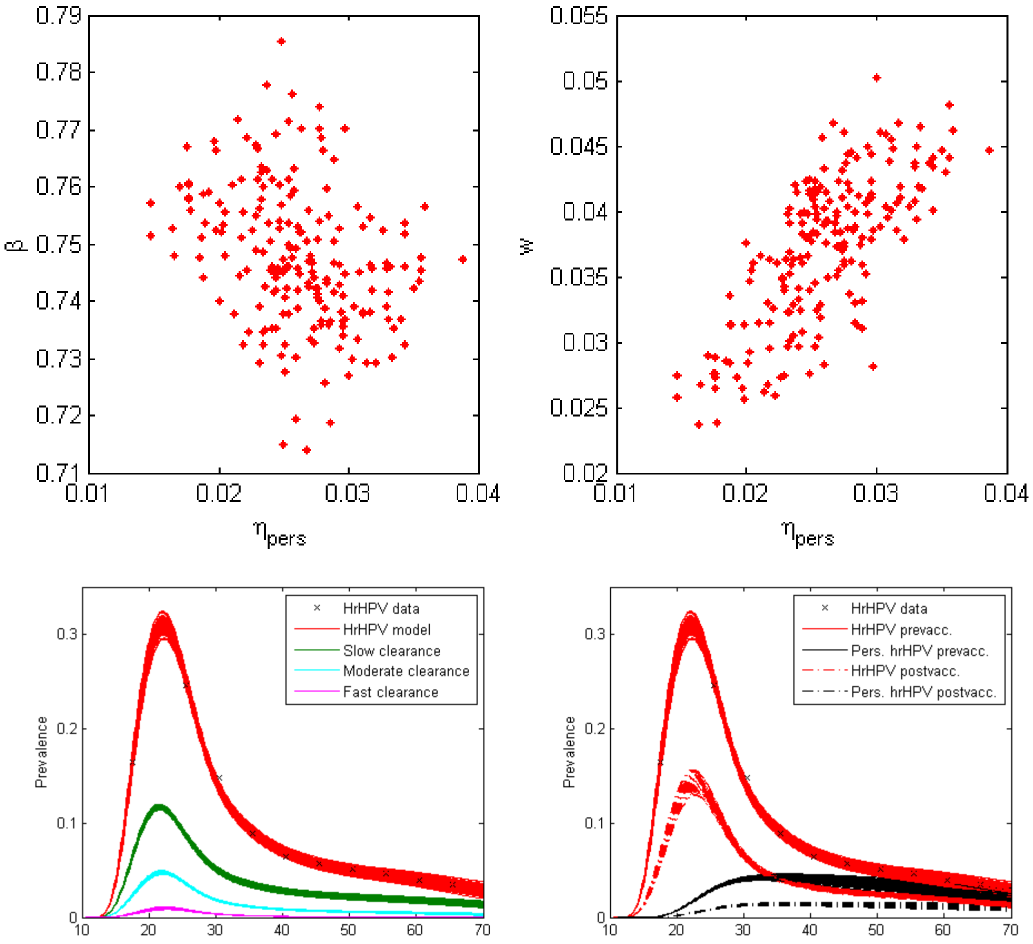

Figure S9. Parameter uncertainty (base-case). The calibrated three model parameters (clearance rate of persistent infections η_{pers}, transmission probability β, and waning rate w of natural immunity) were sampled from the posterior distribution (top panel). For each model parameter triple, the age-specific prevalence were computed before and after the vaccination (bottom panel). The left-side (right-side) figure corresponds to the Figure 4A (4B) of main text.
